# Diagnosing Snapping Sartorius Tendon Secondary to a Meniscal Cyst Using Dynamic Ultrasound Avoids Incorrect Surgical Procedure

**DOI:** 10.1155/2013/813232

**Published:** 2013-10-02

**Authors:** Vipin Asopa, Robert J. Douglas, Jonathan Heysen, David Martin

**Affiliations:** ^1^Orthopaedic Division, Sportsmed SA, 32 Payneham Road, Stepney, SA 5069, Australia; ^2^Medical Division, Sportsmed SA, 32 Payneham Road, Stepney, SA 5069, Australia; ^3^Dr. Jones and Partners, 38 Payneham Road, Stepney, SA 5069, Australia

## Abstract

We describe a case of painful snapping in the medial aspect of the knee of a 40-year-old man, following a knee hyperflexion injury. Dynamic real-time ultrasonography determined that the snapping was due to the distal tendon of sartorius passing over a medial meniscal cyst. The patient subsequently underwent arthroscopic decompression of the cyst instead of an inappropriate hamstring tendon harvest procedure, with complete resolution of symptoms.

## 1. Case Report

Following a hyperflexion injury of the left knee, a 40-year-old male presented to the clinic with difficulty in sleeping because of a painful snapping sensation during flexion and extension of the knee joint. Examination revealed a swelling over the medial joint line, over which a tendon could be felt snapping during flexion and extension of the knee.

**Figure 1:**
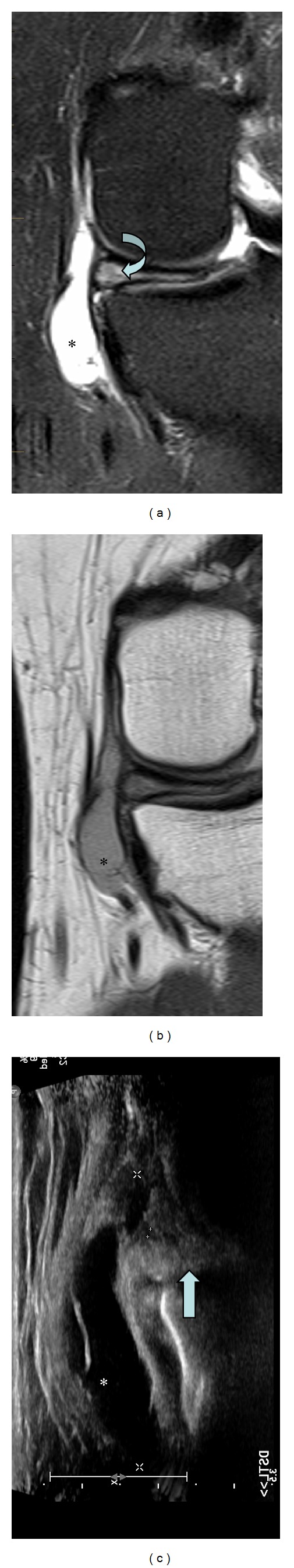
MRI and ultrasound demonstrate the meniscal cyst: (a) coronal fat-saturated proton density MRI. Curved arrow: intrameniscal tear, *parameniscal cyst; (b) coronal proton density MRI. *Parameniscal cyst; (c) ultrasound medial joint line. Straight arrow: horizontal cleavage plane tear medial meniscus, *parameniscal cyst.

**Figure 2:**
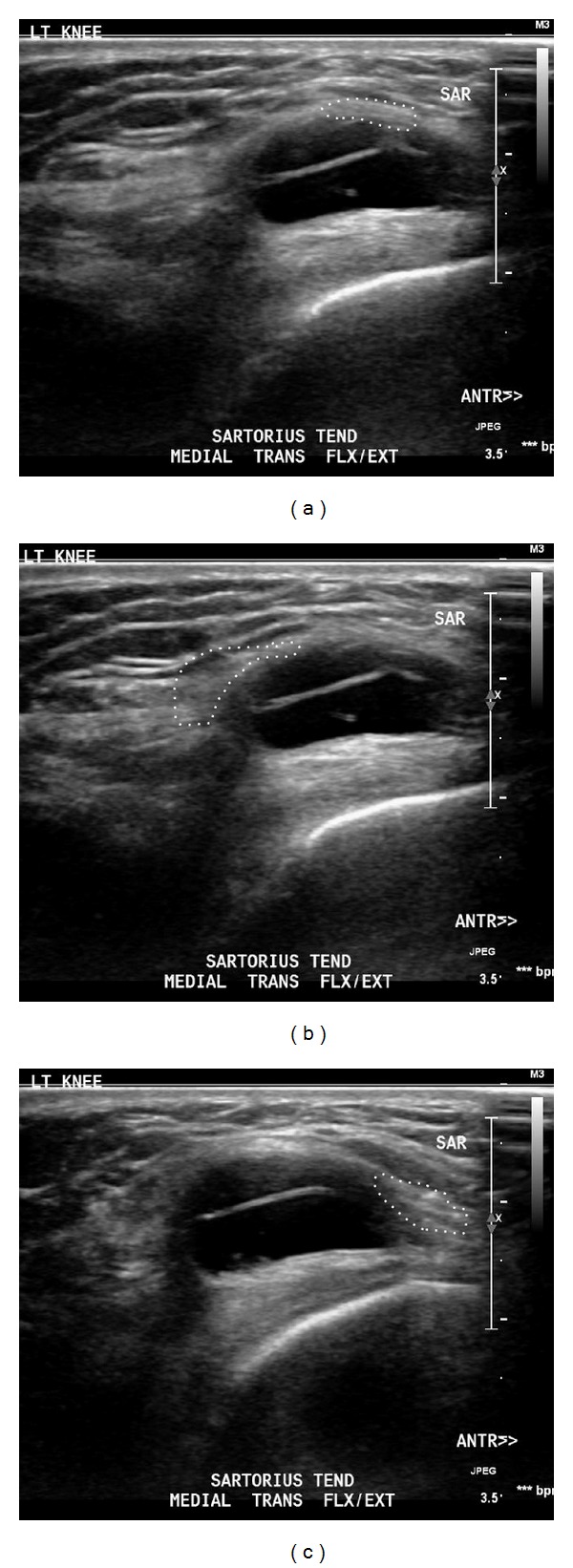
A series of dynamic ultrasound images taken in knee flexion/extension cycle, showing position of distal sartorius musculotendinous junction passing over parameniscal cyst; a palpable “snapping sensation” was felt through the transducer: (a) neutral extension; (b) in semiflexion; (c) extension with active quadriceps contraction; note that the tendon of sartorius passes forwards.

**Figure 3:**
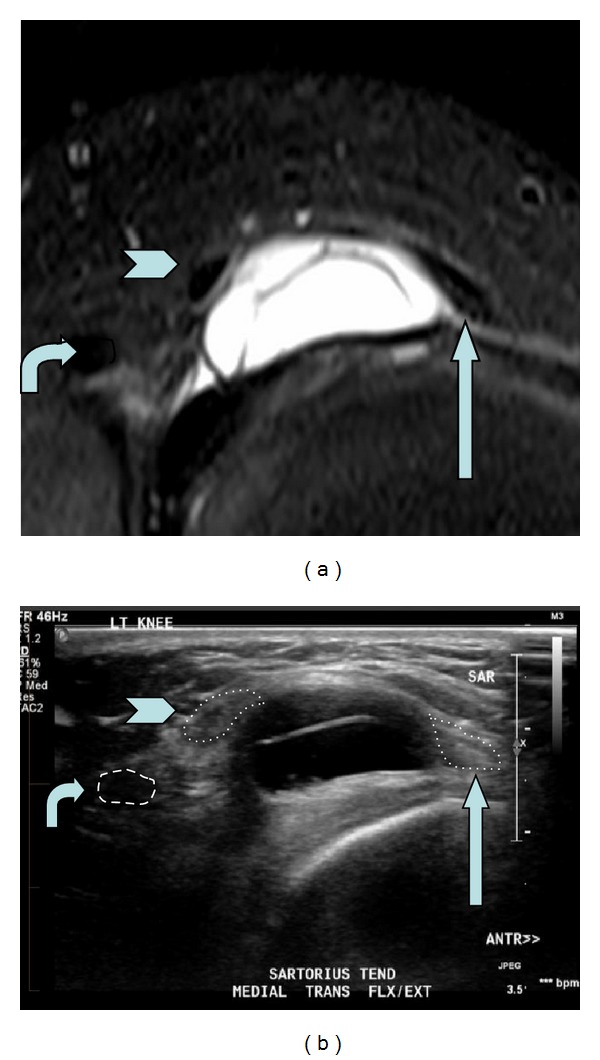
Comparison of (a) fat-saturated proton density MRI and (b) ultrasound left knee. Straight arrow: sartorius tendon; chevron: gracilis tendon; curved arrow: semitendinosus, with sartorius and gracilis “splayed apart” by parameniscal cyst.

Magnetic resonance imaging (MRI) of the knee demonstrated a 20 mm lobulated medial parameniscal cyst, but it was unable to confirm the cause of snapping (Figures 1(a) and 1(b)). Dynamic real-time ultrasonography demonstrated both a meniscal cyst and a meniscal tear (Figure 1(c)). With the knee in neutral extension, ultrasonography showed that the sartorius tendon was anterior to the cyst, with the tendon of gracilis lying posteriorly (Figure 2(a)). On flexion of the knee, the sartorius tendon snapped over and came to lie posterior to the cyst to sit at the anterior margin of gracilis (Figure 2(b)). On extension to neutral with active quadriceps contraction, the sartorius tendon moved rapidly forwards and over the cyst, accompanied by a painful snapping sensation (Figure 2(c)).  Figure 3 contrasts the findings of the fat-saturated proton density MRI with ultrasound of the left knee.

**Figure 4:**
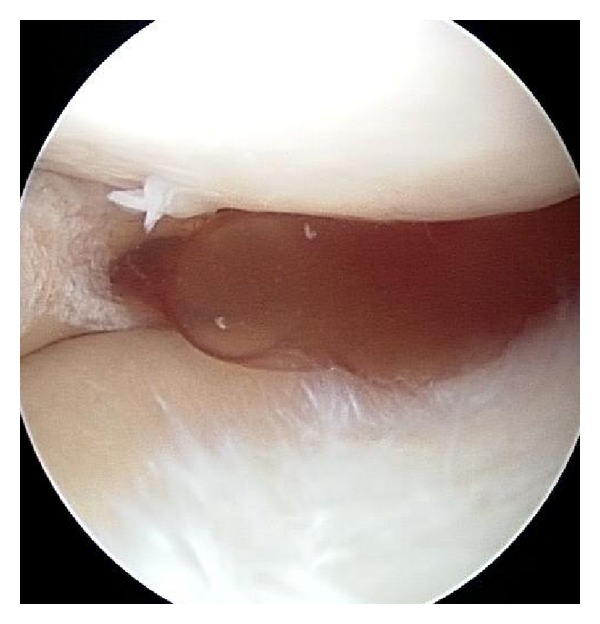
Arthroscopy revealed a meniscal cyst that was decompressed under direct vision.

The patient underwent arthroscopy where a large meniscal cyst was seen (Figure 4). The cyst was arthroscopically decompressed, with complete resolution of symptoms. 

## 2. Discussion

Snapping around the medial aspect of the knee joint can be a diagnostic dilemma. *Pes anserinus* syndrome is a previously described condition caused by snapping of the tendons of semitendinosus and gracilis over the tibial condyle [1–3]. It is thought to be due to deficiency of accessory bands between the tendons and the gastrocnemius fascia [4], and it is treated by surgical release or harvest of the hamstring tendons [5]. Snapping around the knee joint related to the sartorius tendon has been described in two cases; Jain et al. [6] described the condition to be due to movement of the tendon over a bursa that was associated with an anatomically unusual saphenous nerve, and in Nogueira-Barbosa and de Moura Lacerda's case [7], snapping occurred as the tendon passed over the medial femoral condyle.

Although there is a report of the knee joint itself snapping due to a synovial cyst located near the patella-femoral joint (PFJ) which developed as a result of minor knee trauma [8] and despite the meniscal cysts being relatively common [9], there are no previous reports of a meniscal cyst causing a snapping sartorius tendon.

In this case, we describe a syndrome caused by the sartorius tendon snapping over a meniscal cyst. The diagnosis was not possible by MRI alone, and dynamic real-time ultrasound was necessary to direct the correct surgical treatment. Dynamic ultrasound is a quick, cheap, and accurate modality useful in the investigation of the snapping knee tendon, and can benefit the patient by allowing targeted surgical intervention.
